# Supplementary material for: Hyperactive immature state and differential CXCR2 expression of neutrophils in severe COVID-19
Source: Life Sci Alliance. 2022 Dec 13;6(2):e202201658. doi: 10.26508/lsa.202201658 (PMC9748722; doi:10.26508/lsa.202201658)
Supplement: Supplementary file 1 [file LSA-2022-01658_TableS1.docx]

**Supplemental Table 1**

|  | **Healthy controls (n=4)** | **Severe COVID-19 (n=3)** |
| --- | --- | --- |
| **Demographic and clinical data** | | |
| Age, years median ± standard deviation | 27.9 ±6.69 | 54±6.08 |
| Sex, M/F | 2/2 | 3/0 |
| Intensive/high care admission (%) | n/a | 33.3% |
| **Comorbidities** | | |
| Diabetes | 0/4 | 0/3 |
| Heart disease | 0/4 | 0/3 |
| Chronic kidney disease | 0/4 | 0/3 |
| **Sample timeline** median ± standard deviation | | |
| Days since symptom onset | n/a | 18±1.15 |
| Days since hospitalisation | n/a | 4±2 |
