# Supplementary material for: Hyperactive immature state and differential CXCR2 expression of neutrophils in severe COVID-19
Source: Life Sci Alliance. 2022 Dec 13;6(2):e202201658. doi: 10.26508/lsa.202201658 (PMC9748722; doi:10.26508/lsa.202201658)
Supplement: Supplementary file 2 [file LSA-2022-01658_TableS2.docx]

Supplemental Table 2

| **Target-fluorophore** | **clone** | **company** | **Product code** |
| --- | --- | --- | --- |
| CD63 Brilliant violet 421 | H5C6 | Biolegend | 353030 |
| CXCR4 Brilliant violet 605 | 12G5 |  | 306522 |
| CD14 Brilliant violet 785 | M5E2 |  | 301840 |
| CD16 FITC | 3G8 |  | 302006 |
| CD10 PE | HI10a |  | 312204 |
| CXCR2 PE | 5E8 |  | 320722 |
| CD62L PE Cy7 | DREG-56 |  | 304822 |
| CD15 Alexafluor 700 | HI98 |  | 301920 |
| CD66b APC | G10F5 |  | 305118 |
| CD177 APC-Cy7 | MEM-166 |  | 315810 |
| CD101 PER-CP Cy5.5 | BB27 |  | 331015 |
| Streptavidin PER-CP Cy5.5 | N/A |  | 405214 |
| Streptavidin 711 |  |  | 405241 |
| Zombie Aqua |  |  | 423101 |
