# Supplementary material for: Hyperactive immature state and differential CXCR2 expression of neutrophils in severe COVID-19
Source: Life Sci Alliance. 2022 Dec 13;6(2):e202201658. doi: 10.26508/lsa.202201658 (PMC9748722; doi:10.26508/lsa.202201658)
Supplement: Supplementary file 4 [file LSA-2022-01658_TableS3.docx]

**Supplemental Table 3**

| Target | Primer | Sequence |
| --- | --- | --- |
| CXCR2 | Forward | AGCTCTGACTACCACCCAAC |
|  | Reverse | GCTGGGCTTTTCACCTGTAG |
| Actin β | Forward | AGAGCTACGAGCTGCCTGAC |
|  | Reverse | CGTGGATGCCACAGGACT |
